# Effect of hydrothermal pretreatment on the structural changes of alkaline ethanol lignin from wheat straw

**DOI:** 10.1038/srep39354

**Published:** 2016-12-16

**Authors:** Xue Chen, Hanyin Li, Shaoni Sun, Xuefei Cao, Runcang Sun

**Affiliations:** 1 Beijing Key Laboratory of Lignocellulosic Chemistry, Beijing Forestry University, Beijing 100083, China

## Abstract

Due to the enormous abundance of lignin and its unique aromatic nature, lignin has great potential for the production of industrially useful fuels, chemicals, and materials. However, the rigid and compact structure of the plant cell walls significantly blocks the separation of lignin. In this study, wheat straw was hydrothermally pretreated at different temperatures (120–200 °C) followed by post-treatment with 70% ethanol containing 1% NaOH to improve the isolation of lignin. Results demonstrated that the content of associated carbohydrates of the lignin fractions was gradually reduced with the increment of the hydrothermal severity. The structure of the lignins changed regularly with the increase of the pretreatment temperature from 120 to 200 °C. In particular, the contents of *β*-*O*-4′, *β*-*β*′, *β*-5′ linkages and aliphatic OH in the lignins showed a tendency of decrease, while the content of phenolic OH and thermal stability of the lignin fractions increased steadily as the increment of the pretreatment temperature.

As we know, fossil resources have been widely used as fuels, chemicals, and materials for decades. However, the excessive consumption of the non-renewable fossil fuels has caused serious energy crisis and environmental problem. As a renewable and unique carbon-containing resource, biomass is considered as the most important alternative to fossil resources for providing chemicals and materials in the future^1^. Lignocellulosic materials are the most abundant and green biomass available in the world, the utilization of which has drawn worldwide attention^2^,^3^,^4^.

Lignocellulosic materials are mainly composed of cellulose, hemicelluloses, and lignin. Among the three components, lignin is the most abundant aromatic compound in nature that consists of phenylpropane units with various types of linkages. The aromatic nature of lignin presents great potential for the production of industrially useful fuels, chemicals, and materials^5^. Additionally, lignin is considered to be the major barrier to the enzymatic hydrolysis of cellulose for bioethanol production. Therefore, removing lignin from the lignocellulosic raw materials is not only favorable to the valorization of lignin, but also helpful in reducing the recalcitrance of lignocellulose for enzymatic attack. For this purpose, various pretreatment technologies have been developed to improve the removal of lignin from the compact structure of plant cell walls and enhance the digestibility of cellulose by enzymes for bioethanol production^6^.

Generally, pretreatments for lignocellulosic materials can be roughly divided into chemical, physical, physical-chemical and biological methods^7^. Various pretreatment technologies, such as milling, microwave, steam explosion, ammonia fiber explosion (AFEX), irradiation, supercritical CO_2_, alkaline hydrolysis, hydrothermal pretreatment (HTP), organosolv process, have been extensively explored to process different biomasses for cellulosic ethanol production^3^. Among these pretreatments, HTP is an environmentally friendly process because of its outstanding advantages, such as no catalyst requirement, less corrosion, high energy efficiency and so on^8^. The hydrothermal pretreatment process can increase the biomass surface area and decrease the crystallinity of cellulose and thus improve the accessibility of cellulose to enzymes^9^. Furthermore, HTP is also conducive to the dissolution of hemicelluloses and the isolation of lignin in subsequent extraction process^10^. It has been reported that the combination of HTP and NaOH-delignification could remove up to 86% of lignin in wheat straw^11^. Additionally, in consideration of the high efficiency of alkali in disrupting the plant cell walls and the good solubility of lignin in ethanol, a combined process based on hydrothermal pretreatment and alkaline ethanol extraction is considered as a promising method for utilization of lignocellulose with high efficiency. In particular, the hydrolysate obtained in the hydrothermal pretreatment process containing high content of the degraded hemicelluloses derivatives can be used to produce monosaccharides, xylo-oligosaccharides, furfural, etc. The extracted lignin fractions can be applied as feedstocks for the production of high value-added chemicals or materials. It has been reported that lignin showed great potential in various applications, such as dispersant, adhesive, surfactant and water reducer, etc^12^,^13^. The solid residue with high content of cellulose can be further used to prepare bioethanol. Therefore, the utilization efficiency of the lignocellulosic materials can be improved significantly.

Wheat straw is an abundant agriculture residue worldwide. However, it is often burned or discarded in a low value-added utilization method due to the lack of effective use pattern. As a kind of grass, wheat straw possesses much loose structure than wood, which shows great potential for the isolation of lignin and the production of bioethanol. In this work, wheat straw was firstly hydrothermally pretreated under selected conditions and then subsequently post-treated with alkaline ethanol solution to improve the separation of lignin fractions. The structures of the obtained lignins were thoroughly characterized by high performance anion exchange chromatography (HPAEC), gel permeation chromatography (GPC), Fourier transform infrared (FT-IR), carbon-13 nuclear magnetic resonance spectroscopy (^13^C NMR), quantitative two-dimensional heteronuclear single quantum coherence (2D-HSQC) and ^31^P NMR spectroscopy. In addition, thermogravimetric analysis (TGA) was also performed to unveil the relationship between structural features and thermal behaviors. The relationships between physicochemical properties of the isolated lignins and hydrothermal pretreatment conditions were also discussed.

## Results and Discussion

### Yields of the Isolated Lignins and the Content of Associated Carbohydrates

The yields of the isolated lignins (based on the initial weight of the dewaxed wheat straw) and the content of associated carbohydrates (based on the lignin content in the dewaxed wheat straw) are listed in Table 1. As can be seen, the yield of lignin (5.80–12.93%) extracted from the hydrothermally pretreated wheat straw was much higher than that of a lignin sample obtained from the wheat straw without HTP (AL, 3.87%) and the lignin yield increased steadily with the increment of the HTP severity. It suggested that the HTP was beneficial to the following isolation of lignin fraction from wheat straw. Furthermore, it was observed that all the lignin fractions contained relatively low content of associated carbohydrates, which decreased dramatically from 3.09% to 0.06% with the increase of HTP temperature from 120 to 200 °C. In particular, only 0.06% of carbohydrates were detected in L_200_ due to the degradation and removal of hemicelluloses under the harsh hydrothermal condition. The probable reason for the above results may be that the HTP could break the tight cell wall structure and partial chemical bonds between lignin and hemicelluloses, resulting in the easy separation of lignin from the cell walls^14^,^15^.

### Molecular Weight Distribution

To study the effect of the HTP of wheat straw on the molecular weight of the isolated lignin fractions, the weight-average (*M*_w_) and number average (*M*_n_) molecular weights and the polydispersity (*M*_w_/*M*_n_) of the isolated lignins were analyzed by GPC. The results are shown in Table 2. The *M*_w_ value of the lignin fraction obtained from the HTP samples gradually decreased from 2770 g/mol to 1560 g/mol as the HTP temperature increased from 120 to 200 °C. Meanwhile, the *M*_w_ values of the lignin fractions (L_120_, L_140_ and L_160_) obtained from the wheat straw with mild HTP were slightly higher than that of AL (2180 g/mol). It suggested that the mild HTP process was helpful to the decomposion of the cell walls and the release of the lignin fractions with high molecular weights in the following alkaline ethanol extraction. However, the *M*_w_ values of the L_180_ and L_200_ were obviously lower than that of AL, implying that partial linkages between lignin units might be broken and the lignin was degraded into smaller molecules under such severe HTP conditions. Additionally, all the lignin fractions showed relatively narrow molecular distributions (1.54–1.73).

### FT-IR Spectra Analysis

The FT-IR spectra of the lignin fractions obtained from the wheat straw with and without HTP are shown in Fig. S1. Obviously, the typical absorptions at 1605, 1512 and 1427 cm^−1^ arise from the aromatic skeletal vibrations of lignin, indicating that the basic aromatic structure of lignin was not severely disrupted during the HTP and following alkaline ethanol extraction process. The intensity of the absorbance at 1458 cm^−1^ (C–H deformation of –CH_2_– and –CH_3_ groups) decreased from L_120_ to L_200_, suggesting that the content of methoxy groups was decreased with increasing HTP severity as a result of demethoxylation reactions. The absorption at 1342 cm^−1^ (C-O stretching of the S ring breathing) presented a higher intensity than that of the reference band at 1265 cm^−1^ (C-O vibration of the G ring) in L_120_, but the intensity of the band at 1342 cm^−1^ was lower than that at 1265 cm^−1^ in L_160_ and L_200_. It might be related to the demethoxylation reactions of S-type lignin units under the harsh pretreatment conditions, which led to the decrease of the S-type lignin units and the elevation the content of G-type lignin units^16^,^17^. The band at 1155 cm^−1^ observed in all of the lignin spectra is assigned to the antisymmetric C-O stretching of ester groups, suggesting that a portion of ester bonds were remained in the isolated lignins. A band at 1119 cm^−1^ derived from the aromatic in-plane C-H bending deformation of S-type lignin was also observed in all spectra. The intensity of this band decreased with the increment of the HTP temperature from 120 to 200 °C, indicating the reduction of S-type lignin caused by demethoxylation. This result was also supported by the NMR measurements presented afterwards. In addition, it was observed that the intensity of the peak at 1705 cm^−1^ (C = O stretching in conjugated ketone, carbonyl and ester groups) significantly increased with the increment of the HTP severity. It was probably due to the fact that the elimination of water from the benzylic position led to the formation of a carbocation, and then the cleavage of *β*-*O*-4′ linkages gave rise to the formation of Hibbert ketones under severe pretreatment condition^14^,^18^,^19^,^20^.

### Quantitative ^13^C NMR Spectra of the Isolated Lignins

**Figure 1:**
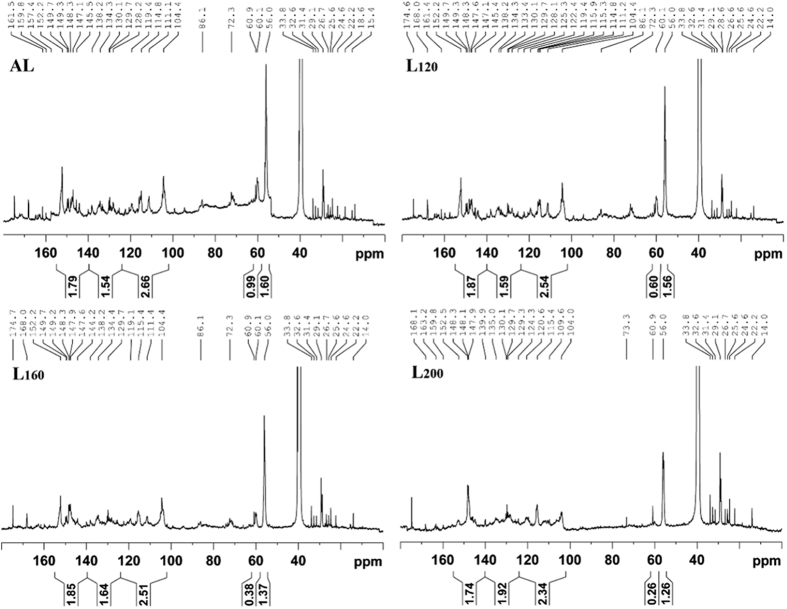
^13^C NMR spectra of the lignin fractions.

The quantitative ^13^C NMR spectra of AL, L_120_, L_160_, and L_200_ are shown in Fig. 1. The major signal assignments and the quantification results of the signals of the lignins are illustrated in Table S1. The carbonyl resonances from esters may contribute to signal at 174.6 ppm, which increased with the increment of HTP temperature. This implied that the majority cleavage of alkoxy side chain may be occurred or large quantities of alkoxy side chain had been modified during the HTP process, such as transformations of alkoxy groups into carbonyl groups (ketones, aldehydes) or carboxylic acid groups^21^.

Signals between 104.4 and 168.2 ppm are attributed to the aromatic region of lignin. The S units signals were observed at 152.2 (C-3/C-5, etherified), 147.1 (C-3/C-5, nonetherified), 138.2 (C-4, etherified), 134.3 (C-1, etherified), and 104.4 ppm (C-2/C-6), while the G units gave signals at 149.7 (C-3, etherified), 145.5 (C-4, nonetherified), 134.3 (C-1, etherified), 119.4 (C-6, etherified), 114.8 (C-5, nonetherified), and 111.1 ppm (C-2, nonetherified). The signals at 128.2 (C-2/C-6) and 115.3 ppm (C-3/C-5, nonetherified) are related to the H units in lignins. These signals indicated that the lignins obtained from wheat straw can be classified as SGH-lignin, which is a typical grass lignin. Additionally, the content of aromatic C-O and aromatic C-H structures in lignins was reduced, while the amount of aromatic C-C structures increased with the increment of HTP intensity. This is likely attributed to the replacement of C-H bonds with C-C bonds during the condensation reaction. Generally, the condensation of lignin occurs simultaneously with its depolymerization during the fractional isolation of lignin. The condensation of lignin tends to form more C-C linkages with the elimination of partial C-H and C-O linkages^21^.

Signals between 50 and 86 ppm are attributed to the interunit linkages of lignin. Signals detected at 86.1 (S units)/84.6 (G and H units), 72.3, and 60.1 ppm are corresponding to the C-*β*, C-*α*, and C-*γ* position of *β*-*O*-4′ linkages, respectively. The signal at 56.0 ppm is assigned to the −OCH_3_ groups in G and S units. The signals of *β*-*O*-4′ linkages and −OCH_3_ groups gradually decreased with the increase of HTP temperature, suggesting that both de-etherification and demethoxylation occurred during the HTP process. The signals between 57.0 and 103.0 ppm, related to the associated polysaccharides, were almost absent in the spectra of the extracted lignins, indicating that a large proportion of linkages between lignin and polysaccharides have been broken as confirmed by the sugar analysis. Additionally, the signal intensity of esterified *p*-coumaric acid (PCA, 129.7 ppm) decreased with the increase of hydrothermal temperature. In particular, the PCA signals were hardly observed as the HTP temperature increased to 200 °C. Meanwhile, the etherified ferulic acid (FA) was detected with a weak signal at 111.1 ppm corresponding to its C-2 position. The variations of these signals suggested that the cleavage of both PCA and FA occurred under the given HTP conditions.

### 2D-HSQC NMR Spectral Analysis

**Figure 2:**
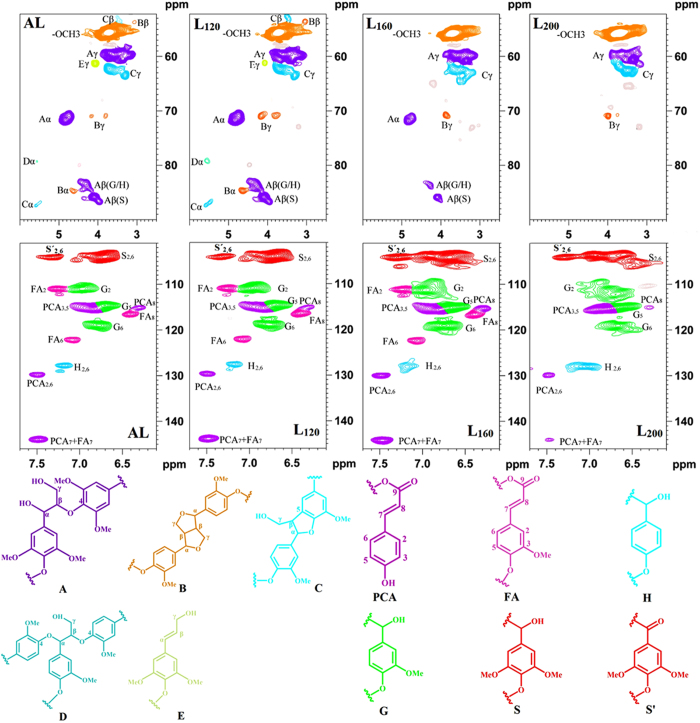
2D-HSQC spectra and the main structures of the lignins.

To obtain the detailed structural information of the lignin samples, 2D-HSQC NMR technique was applied to analyze the lignins. The HSQC spectra of the lignins are presented in Fig. 2. The assignments for cross-signals in side-chain and aromatic regions are shown in Table S2 according to previous literatures^22^,^23^,^24^,^25^. In the side-chain region, cross-signals of various substructures linked by carbon-carbon and ether bonds can be observed. *β*-*O*-4′ aryl ether (substructure A) signals appeared at δ_C_/δ_H_ 71.7/4.82 and 59.6/3.61 are corresponding to its C_*α*_-H_*α*_ and C_*γ*_-H_*γ*_ correlations, respectively. The C_*β*_-H_*β*_ correlations observed at δ_C_/δ_H_ 85.8/4.09 and 83.7/4.28 are assigned to the *β*-*O*-4′ structure linked to S and G/H lignin units, respectively. The interunit *β*-*O*-4′ linkages appeared to be the most abundant linkages of the wheat straw lignin. In addition to the abundant *β*-*O*-4′ linkages, signals for resinol (*β*-*β*′, B) substructures also appeared in the spectra in noticeable amounts with their C_*α*_-H_*α*_, C_*β*_-H_*β*_, and double C_*γ*_-H_*γ*_ correlations at δ_C_/δ_H_ 84.8/4.64, 53.6/3.06, and 71.0/3.79 and 4.16, respectively. Phenylcoumaran (*β*-5′, C) substructures were present in low amounts, and their signals for C_*α*_*-*H_*α*_, C_*β*_-H_*β*_ and C_*γ*_-H_*γ*_ correlations occurred at δ_C_/δ_H_ 87.4/5.58, 53.5/3.45 and 62.2/3.70, respectively. In addition, *α*-*O*-4′ aryl ether (substructure D, δ_C_/δ_H_ 79.3/5.58) and *p*-hydroxycinnamyl alcohol end groups (substructure E, δ_C_/δ_H_ 61.2/4.09) were also detected in the side-chain regions.

In the aromatic region, signals from S, G, and H units could be observed clearly. The C_2,6_-H_2,6_ correlations of S and C_*α*_-oxidized S units (S′) were observed at δ_C_/δ_H_ 104.0/6.69 and 104.1/7.31, respectively. The C_2,6_-H_2,6_ signal of H units was observed at δ_C_/δ_H_ 128.3/7.21, while the G units presented various correlations for C_2_-H_2_ (δ_C_/δ_H_ 111.0/6.97), C_5_-H_5_ (δ_C_/δ_H_ 114.6/6.71), and C_6_-H_6_ (δ_C_/δ_H_ 118.9/6.77). In addition, the signals for PCA substructures were observed at δ_C_/δ_H_ 129.7/7.50, 115.4/6.81, 144.0/7.48 and 115.1/6.30 corresponding to their C_2,6_-H_2,6_, C_3,5_-H_3,5_, C_7_-H_7_ and C_8_-H_8_ correlations, while the signals for FA substructures were detected at δ_C_/δ_H_ 111.0/7.26, 122.1/7.08, 144.0/7.48 and 116.5/6.40 corresponding to C_2_-H_2_, C_6_-H_6_, C_7_-H_7_ and C_8_-H_8_ correlations.

The relative abundances of the interunit linkages presented in lignins as well as PCA, FA and S/G/H molar ratios were calculated from the 2D HSQC spectra based on a previous computing method^26^,^27^. As shown in Table 3, the main lignin linkages of wheat straw lignin were *β*-*O*-4′ aryl ether linkages, followed by low amounts of phenylcoumaran (*β*-5′), resinol (*β*-*β*′), and *α*-*O*-4′ aryl ether linkages. The content of *β*-*O*-4′ linkages in AL (39.35/100Ar) was slightly lower than that of L_120_ (43.10/100Ar). The phenomenon was probably due to the fact that mild hydrothermal pretreatment condition was conducive to the subsequence fractionation of wheat straw lignin to some extent, resulting in the decrease of cleavage of ether linkages during the alkaline ethanol extraction process. However, the content of *β*-*O*-4′ linkages in AL (39.35/100Ar) was much higher than those of L_160_ (19.54/100Ar) and L_200_ (1.50/100Ar). It indicated that extensive depolymerization reactions occurred under such severe pretreatment condition. Except for the cleavage of *β*-*O*-4′ linkages, comprehensive degradation of other aryl ether linkages (*α*-*O*-4′ linkages) and carbon-carbon linkages (*β*-*β*′ and *β*-5′) was also observed.

Besides the interunit linkages difference among these lignins obtained under different HTP conditions, a notable lignin units difference among the lignins were also found. As shown in Table 3, the relative amount of S units gradually decreased and the relative abundance of G and H units increased with the increment of the HTP temperature from 120 to 200 °C. This phenomenon can be explained by that extra G and H units were formed through the demethoxylation of S and/or G units under the HTP condition. In addition, it was also found that the contents of FA and PCA in L_120_, L_160_ and L_200_ were lower than those in AL, suggesting that partial etherified FA and esterified PCA in lignin were disrupted during the HTP process^22^,^28^.

### ^31^P NMR Spectra Analysis

To further investigate the functional groups of the obtained lignins, the lignin samples (AL, L_120_, L_160_, and L_200_) were analyzed using the quantitative ^31^P NMR technique. The ^31^P NMR spectra of the lignins are illustrated in Fig. S2 and the contents of aliphatic OH, phenolic OH, and carboxylic group of the lignins are listed in Table 4. An increase of phenolic OH content and a decrease of aliphatic OH content were observed as the increment of HTP temperature. The aliphatic OH in the lignin fractions extracted from the hydrothermally pretreated wheat straw decreased significantly from 2.97 to 0.91 mmol/g as compared to that of the AL (3.36 mmol/g) obtained without pretreatment. It suggested that the aliphatic OH groups were oxidized and modified gradually over the HTP process. Similar results on ethanol organosolv lignin extracted from Miscanthus × giganteus were also reported by Ei Hage *et al*.^29^. Additionally, the content of the total phenolic OH (1.45–2.23 mmol/g) in the lignins isolated from the hydrothermally pretreated wheat straw was higher than that of AL (1.33 mmol/g). The increase of S-type phenolic OH and G-type phenolic OH was related to the cleavage of aryl ether linkages, while the increment of condensed G-type phenolic OH was attributed to the occurrence of more condensation reactions caused by the increase of the HTP temperature. The observed raise of phenolic OH in condensed lignin units was also confirmed by the enhancement of aromatic C-C correlations from the ^13^C NMR analysis.

### Thermogravimetric Analysis

**Figure 3:**
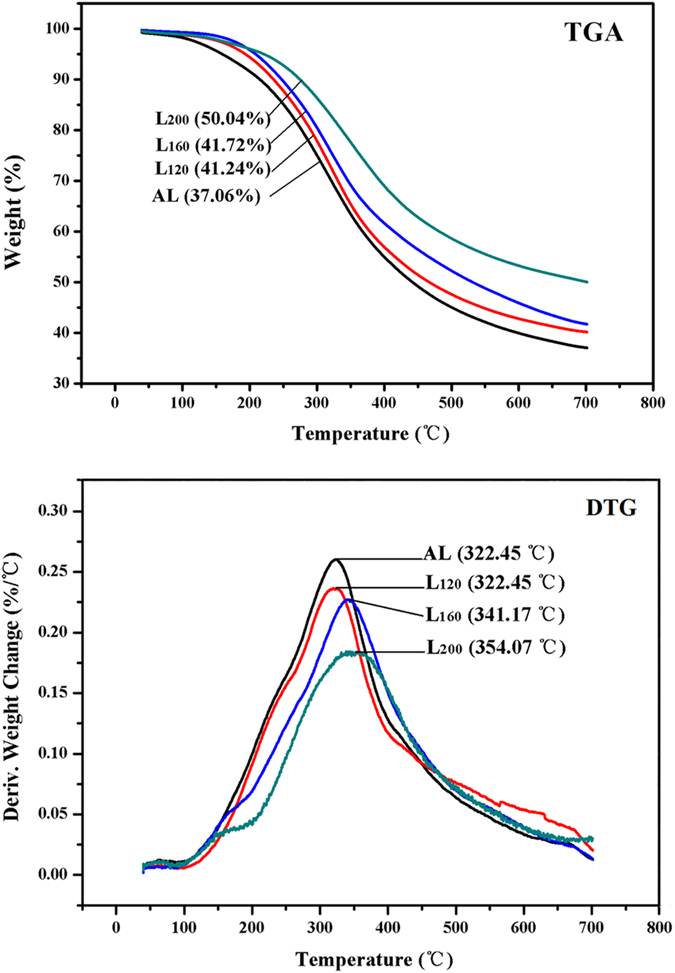
TGA and DTG curves of the lignins.

The thermal stability of lignin is an important parameter for its utilization in thermoplastic or thermosetting materials. The decomposition of lignin is very complex, in which several competing reactions might occur^30^,^31^. Generally, the thermal stability of lignin can be affected by their inherent structures, variable functional groups, and degrees of branching and condensation. To understand the relationship between structural features and thermal behaviors of the isolated lignins, thermogravimetric technique was applied to AL, L_120_, L_160_ and L_200_ in this experiment. Figure 3 shows the TGA and DTG curves of the obtained lignins.

The main degradation temperature range of the lignins was observed between 200 and 700 °C. Generally, the maximum decomposition temperature (T_*M*_) is in line with the temperature of the maximum decomposition rate (V_*M*_) of substrate^32^. As shown in Fig. 3, the T_*M*_ of the lignin obtained with HTP was higher than that of the lignin extracted without HTP, and the T_*M*_ of the isolated lignin increased with the raising HTP temperature. It has been reported that the T_*M*_ of lignin was positively related to its molecular weight^33^. However, this relationship between T_*M*_ and *M*_*w*_ was not found in the present work. The shift of T_*M*_ to higher temperature might be explained by the formation of more stable lignin structure, such as condensed lignin, which was confirmed by the NMR results. Additionally, a large proportion of residual char was observed after calcination at 700 °C. The residual chars were 37.06% and 41.24–50.04% for AL and L_120_-L_200_, respectively. Moreover, the residual char percentage showed an increased trend with the raising HTP severity. The char percentage was positively correlative with the content of the condensed lignin units in lignin uncovered by the ^31^P NMR analysis, indicating the high thermal stability of condensed lignin.

## Conclusion

Wheat straw with and without hydrothermal pretreatment was extracted with alkaline ethanol solution to release the lignin fractions. The yield of the lignin (5.80–12.93%) obtained from the hydrothermally pretreated wheat straw was much higher than that of AL (3.87%) isolated from the wheat straw without hydrothermal pretreatment. The structures and physicochemical properties of the lignins isolated from the hydrothermally pretreated wheat straw changed regularly with the increase of pretreatment temperature from 120 to 200 °C. In particular, the contents of *β*-*O*-4′, *β*-*β*′, *β*-5′ linkages and aliphatic OH in the lignins showed tendency of decrease, while the content of phenolic OH in the lignins increased steadily as the increment of the pretreatment temperature. Moreover, the molecular weight of the lignins was decreased but the thermal stability of the lignins was increased with the raise of the hydrothermal pretreatment severity. It suggested that the demethoxylation, depolymerization and condensation reactions of lignin occurred simultaneously during the hydrothermal pretreatment process. The results presented in this study will be conducive to the production of lignin-based products for the industrial application of lignin.

## Materials and Methods

### Materials

Wheat straw was obtained from Shaanxi province, China. The dried wheat straw was grounded, and the fraction between 40–60 mesh was collected for further experiments. The straw powder was further dewaxed with toluene-ethanol (2:1, v/v) in a Soxhlet apparatus for 6 h and then dried in an oven at 60 °C for 16 h. The dewaxed straw was mainly composed of 41.2% cellulose, 27.7% hemicelluloses, 18.5% lignin (16.6% Klason lignin and 1.9% acid-soluble lignin), and 6.9% ash, according to National Renewable Energy Laboratory’s (NREL) standard analytical procedure^34^. All chemical reagents were analytical grade and used without further purification.

### Hydrothermal Pretreatment (HTP) and Fractionation Process

**Figure 4:**
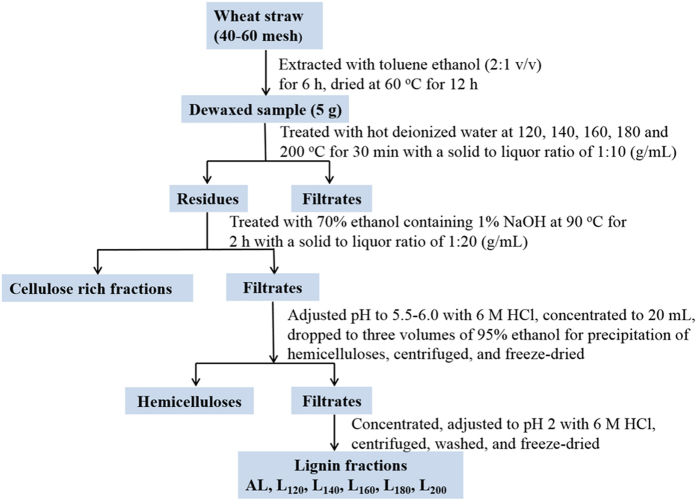
Schematic diagram for fractionating lignin from wheat straw.

The HTP of wheat straw was conducted in a 100 mL batch reactor (Beijing Century Sen Long Instruments Company, Beijing, China). The feedstock 5.0 g was immersed in 50 mL deionized water. Then, the mixture was heated up to desired temperature and maintained at this temperature for 0.5 h under continuous mechanical stirring at 800 rpm. After completion of the reaction, the reactor was cooled down to room temperature by ice water. The hydrothermally pretreated residue was collected by filtration, washed thoroughly with deionized water and freeze-dried. Then, the hydrothermally pretreated residue (2.0 g) was subsequently treated with 70% ethanol solution containing 1% NaOH at 90 °C for 2 h at a solid to liquid ratio of 1:20 (g/mL). The solid residue and liquid stream were separated on a Buchner funnel, washed with deionized water until the filtrate was neutral. The filtrate was collected and adjusted to pH 5.5–6.0 with 6 M HCl and further concentrated to 40 mL on a rotary evaporator under reduced pressure. The concentrated liquid was poured into three volumes of 95% ethanol and further filtrated. The supernatant was further concentrated to about 10 mL and poured into 100 mL of acidic water (pH 2.0 adjusted by HCl) to precipitate lignin. The obtained lignin fractions were freeze-dried and labeled as L_120_, L_140_, L_160_, L_180_, and L_200_ according to the HTP temperature. A lignin sample (AL) fractionated from the wheat straw without hydrothermal pretreatment was also prepared for comparison. All experiments were performed in duplicate, and the average values were given. The schematic diagram of the experimental procedure is shown in Fig. 4.

### Analysis Procedures

The weight-average (*M*_w_) and number-average (*M*_n_) molecular weights of the obtained lignin fractions were determined by GPC with an ultraviolet detector (UV) at 240 nm according to a previous literature procedure^22^. The associated carbohydrates in the lignin fractions were determined by using HPAEC based on a previous report^22^. FT-IR spectra of the lignin fractions were obtained on a Bruker spectrophotometer in the range of 800–4000 cm^−1^ with a resolution of 4 cm^−1^.

NMR spectra were recorded on a Bruker AVIII 400 MHz spectrometer at 25 °C in DMSO-*d*_6_. For the quantitative ^13^C NMR experiments, 140 mg of lignin was dissolved in 0.5 mL DMSO-*d*_6_. To achieve sufficient relaxation in a feasible time, 20 μL of chromium (III) acetylacetonate (0.01 M) was added as a relaxation agent for the quantitative ^13^C NMR spectrum to reduce the relaxation delay according to previous reports^35^,^36^. The quantitative ^13^C NMR spectra were recorded in the FT mode at 100.6 MHz. The inverse gated decoupling sequence (C13IG sequence from Bruker Standard Library) allows quantitative analysis and comparison of signal intensities, which was used with the following parameters: 30° pulse angle; 1.4 s acquisition time; 2 s relaxation delay; 64 K data points, and 30,000 scans. For 2D HSQC NMR spectra, 50 mg lignin was dissolved in 0.5 mL DMSO-*d*_6_. A semiquantitative analysis of the 2D-HSQC contour intensities was performed according to literature methods^37^,^38^. Phenolic hydroxyl, aliphatic hydroxyl and carboxyl groups of the lignin obtained were quantitated by ^31^P NMR spectra^39^,^40^. 20 mg dry lignin was dissolved in 500 μL deuterated chloroform and anhydrous pyridine (1:1.6, v/v) under stirring, followed by addition of 100 μL of chromium (III) acetylacetonate solution (5 mg/mL in anhydrous pyridine and deuterated chloroform 1.6:1, v/v) as a relaxation reagent. Then, 100 μL of cyclohexanol (10.85 mg/mL in anhydrous pyridine and deuterated chloroform 1.6:1, v/v) was added as an internal standard. Finally, the mixture was treated with 100 μL of phosphitylating reagent (TMDP) and transferred into a 5 mm NMR tube for subsequent analysis^39^.

Thermal analysis of the lignin fractions was measured by using TGA and differential thermogravimetric (DTG) analyses on a simultaneous thermal analyzer (DTG-60, Shimadzu, Japan). About 3–5 mg of the lignin sample was heated in an alumina crucible from room temperature to 700 °C at a heating rate of 20 ^o^C/min under nitrogen atmosphere.

## Additional Information

**How to cite this article**: Chen, X. *et al*. Effect of hydrothermal pretreatment on the structural changes of alkaline ethanol lignin from wheat straw. *Sci. Rep.* **6**, 39354; doi: 10.1038/srep39354 (2016).

**Publisher’s note:** Springer Nature remains neutral with regard to jurisdictional claims in published maps and institutional affiliations.

## Supplementary Material

Supplementary Information

## Figures and Tables

**Table 1:** Yields and the content of associated carbohydrates (absolute %, w/w) of the isolated lignins.

	Yield^a^	Glucose	Xylose	Mannose	Arabinose	Total sugars^b^
AL	3.87	0.29	0.10	0.08	0.05	0.52
L_120_	5.80	0.08	0.71	2.28	0.02	3.09
L_140_	5.97	0.17	0.47	1.46	0.04	2.14
L_160_	7.67	0.15	0.69	ND^c^	0.04	0.88
L_180_	8.45	0.18	0.19	ND	0.03	0.40
L_200_	12.93	0.02	0.04	ND	ND	0.06

^a^Based on the initial weight of the dewaxed wheat straw (%, w/w).

^b^Based on the dry percent weight in the isolated lignins (%, w/w).

^c^ND, not detectable.

**Table 2:** Weight-average (*Μ*_*w*_) and number-average (*Μ*_*n*_) molecular weights, and polydispersity (*Μ*_*w*_ */Μ*_*n*_) of the lignin fractions.

	*Μ*_*w*_	*Μ*_*n*_	*Μ*_*w*_/*Μ*_*n*_
AL	2180	1420	1.54
L_120_	2770	1600	1.73
L_140_	2620	1540	1.70
L_160_	2490	1510	1.65
L_180_	1910	1160	1.64
L_200_	1560	970	1.61

**Table 3:** Quantitative characteristics of lignin fractions from 2D-HSQC spectra.

	S/G/H	*β*-*O*-4′	*β-β*′	*β*-5′	*α*-*O*-4′	FA	PCA
AL	46/51/3	39.35	5.83	3.06	1.70	17.25	5.76
L_120_	55/43/2	43.10	5.67	3.46	2.44	13.15	4.16
L_160_	47/50/3	19.54	1.88	0.36	ND^a^	13.10	4.62
L_200_	31/63/6	1.50	ND	ND	ND	ND	1.27

^a^ND, not detectable.

**Table 4:** Quantification of the functional groups (mmol/g) in the lignins by quantitative ^31^P-NMR method.

	Aliphatic OH	Syringyl OH	Condensed Guaiacyl OH	Non-condensed Guaiacyl OH	*p*-Hydroxyphenyl OH	Carboxylic group	Total phenolic OH
AL	3.36	0.16	0.10	0.69	0.38	1.06	1.33
L_120_	2.97	0.29	0.14	0.71	0.31	0.80	1.45
L_160_	2.48	0.43	0.19	0.71	0.24	0.56	1.58
L_200_	0.91	0.77	0.29	0.87	0.31	0.56	2.23
