# Supplementary material for: Effect of hydrothermal pretreatment on the structural changes of alkaline ethanol lignin from wheat straw
Source: Sci Rep. 2016 Dec 16;6:39354. doi: 10.1038/srep39354 (PMC5159836; doi:10.1038/srep39354)
Supplement: Supplementary Information [file srep39354-s1.pdf]

Supporting Information

Effect of hydrothermal pretreatment on the structural changes of alkaline ethanol lignin from wheat straw

Xue Chen, Hanyin Li, Shaoni Sun, Xuefei Cao* & Runcang Sun*

Beijing Key Laboratory of Lignocellulosic Chemistry, Beijing Forestry University, Beijing 100083, China

*Authors to whom correspondence should be addressed.

E-mail: rcsun3@bjfu.edu.cn (Runcang Sun) or caoxuefei@bjfu.edu.cn (Xuefei Cao). Tel.: +86-010-62336903; Fax: +86-010-62336903.

Table S1

The assignment and quantification of the signals of the ^{13}C-NMR spectra (results expressed per Ar) of the lignin fractions.

δ (ppm)	Assignment	AL	L_{120}	L_{160}	L_{200}
158-140	Aromatic C-O	1.79	1.87	1.85	1.74
140-124	Aromatic C-C	1.54	1.59	1.64	1.92
124-102	Aromatic C-H	2.66	2.54	2.51	2.34
62-58.1	*β*-*O*-4′ linkages	0.99	0.60	0.38	0.26
58.1-54.5	$-OCH_3$	1.60	1.56	1.37	1.26

Table S2

Assignments of ^{13}C-^{1}H cross-peaks in HSQC spectra of the lignins.

Lable	δ_C/δ_H (ppm)	Assignments
C_β	53.5/3.45	C_β–H_β in phenylcoumaran substructures (C)
B_β	53.6/3.06	C_β–H_β in *β*-*β′* (resinol) substructures (B)
$–OCH_3$	55.5/3.70	C−H in methoxyls
A_γ	59.6/3.61	C_γ–H_γ in *β*-*O*-4′ substructures (A)
E_γ	61.2/4.09	C_γ–H_γ in *p*-hydroxycinnamyl alcohol end groups (F)
C_γ	62.2/3.70	C_γ–H_γ in phenylcoumaran substructures (C)
B_γ	71.0/3.79-4.16	C_γ–H_γ in *β*-*β′* (resinol) substructures (B)
A_α	71.7/4.82	C_α–H_α in *β*-*O*-4′ units (A)
D_α	79.3/5.58	C_α–H_α in *α*, *β*-diaryl ethers (E)
$A_{\beta(G/H)}$	83.7/4.28	C_β–H_β in *β*-*O*-4′ linked to a G/H unit (A)
B_α	84.8/4.64	C_α–H_α in *β*-*β′* (resinol) substructures (B)
$A_{\beta(S)}$	85.8/4.09	C_β–H_β in *β*-*O*-4′ linked to an S unit (A)
C_α	87.4/5.58	C_α–H_α in phenylcoumaran substructures (C)
$S_{2,6}$	104.0/6.69	$C_{2,6}$–$H_{2,6}$ in syringyl units (S)
$S'_{2,6}$	104.1/7.31	$C_{2,6}$–$H_{2,6}$ in oxidized (C_α=O) S units (S′)
G_2	111.0/6.97	C_2–H_2 in guaiacyl units (G)
G_5	114.6/6.71	C_5–H_5 in guaiacyl units (G)
G_6	118.9/6.77	C_6–H_6 in guaiacyl units (G)
$H_{2,6}$	128.3/7.21	$C_{2,6}$–$H_{2,6}$ in H units (H)
$PCA_{3,5}$	115.4/6.81	$C_{3,5}$–$H_{3,5}$ in *p*-coumaric acid (PCA)
$PCA_{2,6}$	129.7/7.50	$C_{2,6}$–$H_{2,6}$ in *p*-coumaric acid (PCA)
PCA_7	144.0/7.48	C_7–H_7 in *p*-coumaric acid (PCA)
PCA_8	115.1/6.30	C_8–H_8 in *p*-coumaric acid (PCA)
FA_2	111.0/7.26	C_2–H_2 in ferulic acid (FA)
FA_6	122.1/7.08	C_6–H_6 in ferulic acid (FA)
FA_7	144.0/7.48	C_7–H_7 in ferulic acid (FA)
FA_8	116.5/6.40	C_8–H_8 in ferulic acid (FA)

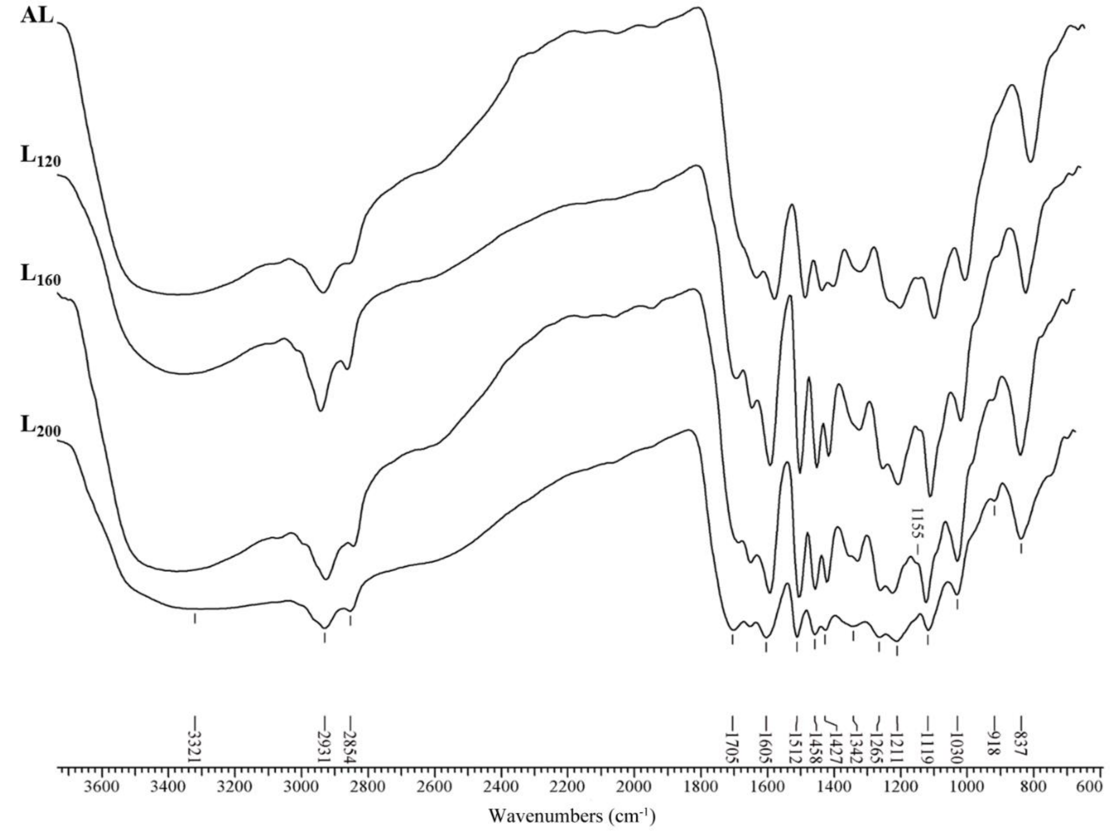

Fig. S1. FT-IR spectra of the lignins.

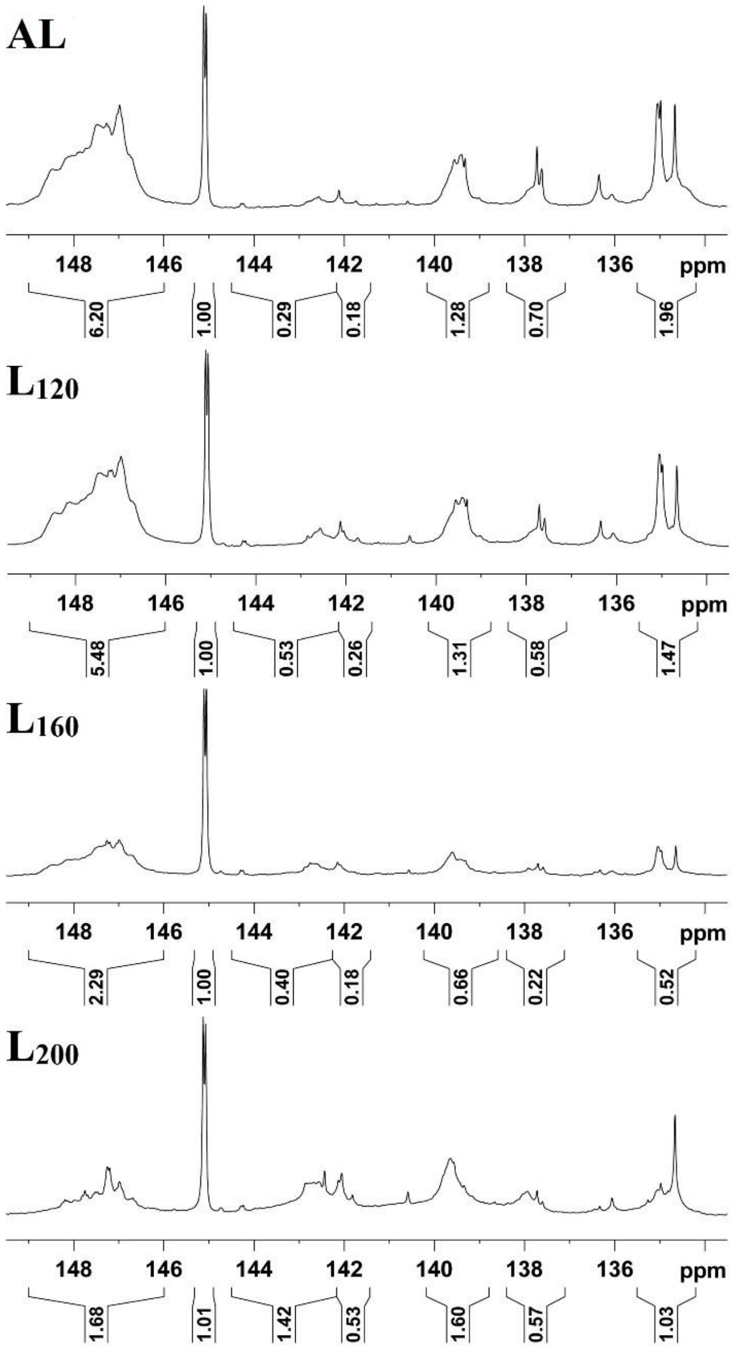

Fig. S2. ^{31}P-NMR spectra of the lignins.
